# Transudative or masked exudative polyserositis in disseminated tuberculosis? A case report

**DOI:** 10.1016/j.amsu.2022.103891

**Published:** 2022-05-31

**Authors:** Bishal Dhakal, Prabhat K.C, Sachin Sapkota, Binaya Subedi, Apshara Acharya, Suvekchya Pandey, Dilip Thapa

**Affiliations:** a Nepalese Army Institute of Health and Sciences, Kathmandu, Nepal; b Maulakalika Hospital Pvt. Ltd., Bharatpur, 10-Chitwan, Nepal

**Keywords:** Tuberculosis (TB), Disseminated TB, Polyserositis, Transudative, Exudative

## Abstract

**Introduction:**

Polyserositis in disseminated tuberculosis (TB) is an uncommon presentation. The exudative nature of effusion in disseminated TB can be masked by presence of malnutrition due TB.

**Case presentation:**

A 24-year-old female, diagnosed with disseminated TB, developed polyserositis with transudative nature of fluid. She was treated with anti-tubercular therapy (ATT).

**Clinical discussion:**

Polyserositis, though an uncommon presentation in disseminated TB, was the clinical manifestation in our case. But transudative nature of the fluid was an unexpected finding. Hypoalbuminemia as a result of malnutrition due to TB was the cause for masking exudative effusion in TB.

**Conclusions:**

Hypoalbuminemia as a result of malnutrition due to TB can be the reason for transudative nature of effusion in polyserositis.

## Highlights

• Polyserositis is an uncommon presentation in disseminated TB.
• The exudative nature of fluid is a common finding in polyserositis due to disseminated TB.
• The transudative nature of the fluid can also be the finding in polyserositis due to disseminated TB.
• Hypoalbuminemia as a result of malnutrition due to TB can lead to transudative polyserositis in disseminated TB.

## 1 Introduction

Tuberculosis (TB) is one of the common infectious diseases caused by *Mycobacterium tuberculosis*. TB has various forms of presentation, common being pulmonary and extra-pulmonary forms. Disseminated TB can be simply defined as lymphohematogenous dissemination of tubercle bacilli in two or more non-contiguous sites [1]. The variety of presentations in disseminated TB makes its diagnosis a difficult one [2,3]. Polyserositis is disseminated TB is an uncommon feature [4].

Disseminated TB is life-threatening in cases of diagnostic delay. The clinical, radiological and histopathological diagnostic pathways have been described in the diagnosis of TB. Due to the non-specific presentation like polyserositis and its unusual nature, diagnosis is often delayed. With the aim and objectives of describing unusual presentations like polyserositis, its transudative nature and reason behind such nature in disseminated TB, we hereby, present a case disseminated TB with polyserositis and its unusual nature in a 24-year-old female.

## 2 Case presentation

A 24-year-old Hindu female, known case of bilateral polycystic kidney disease, presented to medical out-patient department (OPD) with complaint of watery diarrhea for one month. It started with the frequency of 4–5 episodes per day and was non-blood stained. It was associated with malaise, vomiting, abdominal pain, loss of appetite and weight loss. Gradually, it was severe enough to affect her daily activities. There was no history of fever, food poisoning and travel to distant places. She had no prior history of tuberculosis (TB), hypertension and diabetes mellitus. There was also not any relevant surgical and medication history for any disease.

**Fig. 1:**
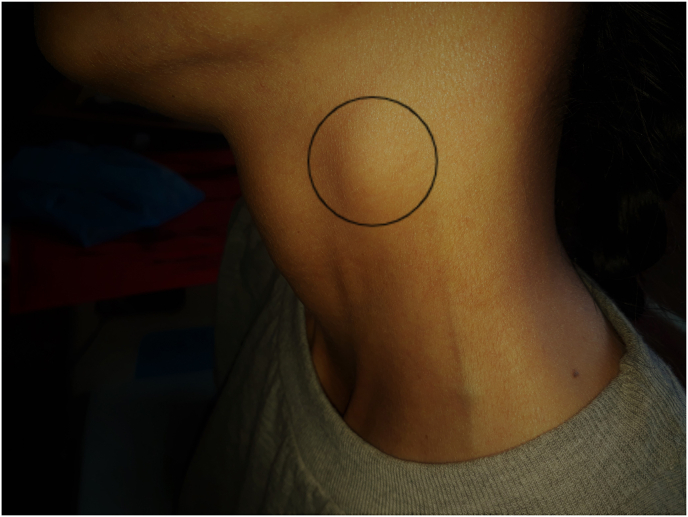
Lateral neck swelling. Legend: Circle showing enlarged cervical lymph node in lateral neck.

On general examination, she was ill-looking, cachectic but conscious and oriented. She had pallor in eyes, tongue and hands and edema in bilateral lower limbs. There were multiple palpable cervical lymph nodes as shown in Fig. 1. At admission, her vital parameters were stable and no abnormality was detected during systemic examination. Later during hospital stay, she had bilateral decreased air entry with conducted sounds and bilateral (right > left) crepitations on chest examination. On per abdomen examination, she had distended abdomen with positive shifting dullness test. The differentials considered at the time of admission were tuberculosis, malabsorption syndrome, irritable bowel syndrome (IBS) and inflammatory bowel disease (IBD).

The laboratory investigations are shown in Table 1.

**Table 1:** Laboratory investigations; TIBC: Total Iron Binding Capacity.

Laboratory tests	Result	Unit	Reference range
Total Leukocytes Count (TLC)	8.7	10^˄^3/μL	4–11
Neutrophil	80	%	40–80
Lymphocyte	13	%	20–40
Hemoglobin	**9.3**	g/dl	13–17
Platelet Count	447	10^˄^3/μL	150–450
Urea	26.8	mg/dl	17–43
Creatinine	0.45	mg/dl	0.7–1.3
Sodium	135	mEq/L	135–145
Potassium	4.0	mEq/L	3.5–5.5
Bilirubin Total	0.8	mg/dl	0.1–1.2
Bilirubin Direct	0.3	mg/dl	0.0–0.2
Alkaline Phosphatase (ALP)	48	U/L	53–128
Alanine Transferase (ALT)	34	U/L	0–35
Aspartate Transferase (AST)	32.7	U/L	0–35
Random Blood Glucose	115	mg/dl	70–140
Prothrombin time (PT)	15.2	seconds	11–13.5
CPK NAC	68	U/L	20–200
CPK MB	17.7	U/L	<35
Troponin I	Negative		
C-Reactive Protein (CRP)	**52.28**	mg/dl	<6.4
Lactate dehydrogenase (LDH)	**276**	U/L	140–280
Serum ferritin	**434.5**	μg/L	20–250
Serum iron	**12.96**	μg/dl	50–100
TIBC	**112**	μg/dl	228–428

**Fig. 2:**
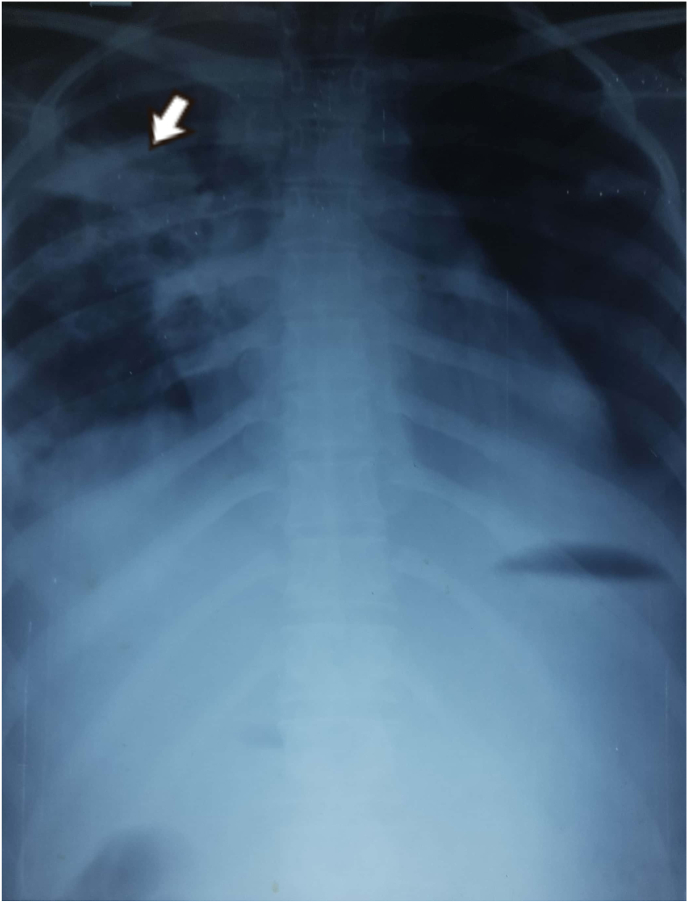
Chest X-ray PA view. Legend: Arrow showing heterogenous opacity with air bronchogram (consolidation) with absence of miliary shadowing.

**Fig. 3:**
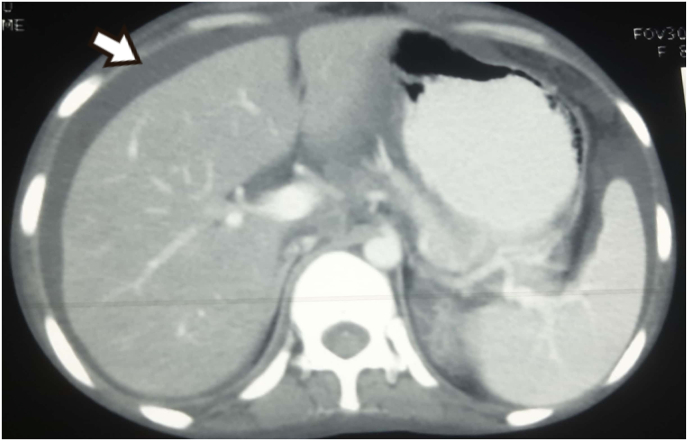
CECT chest and abdomen. Legend: Arrow showing pleural effusion.

**Fig. 4:**
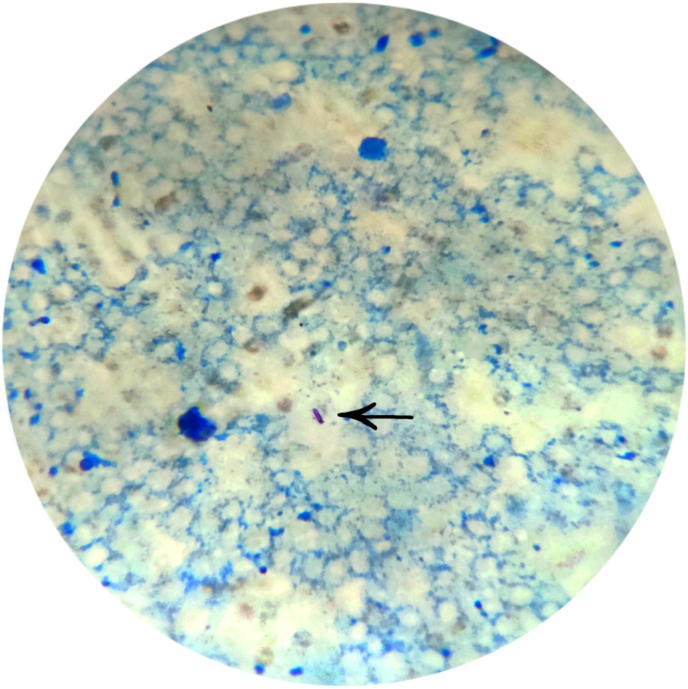
ZN stain of FNAC specimen. Legend: Arrow showing AFB in blue background under 1000× magnification.

**Fig. 5:**
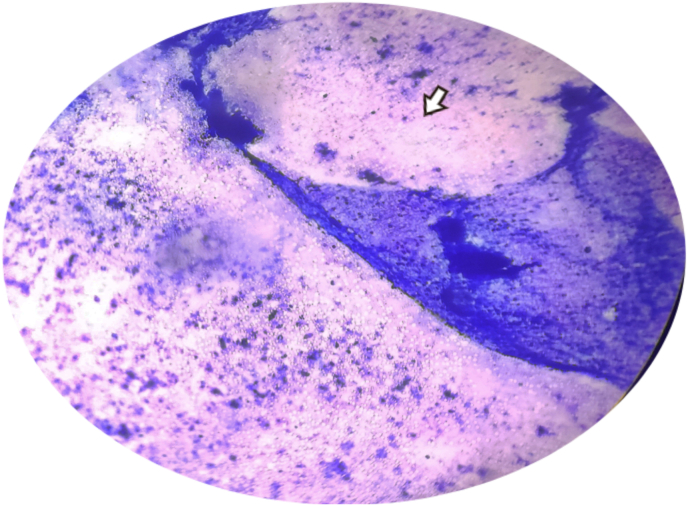
FNAC of cervical lymph node. Legend: Arrow showing caseating necrosis with dirty background.

During etiological workup, the tropical fever profile was negative. During the anemia workup, peripheral blood smear (PBS) had findings of microcytic hypochromic anemia. The chest X-ray showed consolidation, bilateral pleural effusion with no miliary shadows as in Fig. 2. Ultrasonography of abdomen and pelvis revealed bilateral pleural effusion with gross ascites. There was evidence of pleural and pericardial effusion with normal ejection fraction on contrast enhanced computed tomography (CECT) of chest and 2D-echocardiography respectively. The CECT (chest, abdomen and pelvis) also showed multifocal consolidation in lungs with surrounding ground glass opacities in left lung suggesting tuberculosis (TB) etiology. The CECT chest and abdomen showing pleural effusion is shown in Fig. 3. Likewise, multiple enlarged mesenteric and retroperitoneal lymph nodes with gross ascites and peritoneal thickening were also seen. All these findings were suggestive of disseminated TB. Mantoux test revealed no induration after 48 hours. Fine needle aspiration cytology (FNAC) of right cervical lymph node was done. It revealed tubercular lymphadenitis with acid fast bacilli (AFB) seen in Ziehl-Neelsen (ZN) stain as shown in Fig. 4, Fig. 5. USG guided aspiration of ascitic, pleural and pericardial fluid was done and sent for fluid analysis. The Gene Xpert of pleural fluid detected *Mycobacterium tuberculosis (*MTB) without rifampicin resistance. All these investigations were done to find out the etiology for symptoms and signs at the time of presentation and polyserositis that was developed later during the hospital stay. The fluid analysis is shown in Table 2.

**Table 2:** Fluid analysis; ADA: Adenosine deaminase.

Parameters	Serum	Ascitic	Pleural	Pericardial
Protein (g/dl)	**4.81** [[6], [7], [8]]	0.92 (0–8.5)	**0.63** [[6], [7], [8]]	**0.92** [[6], [7], [8]]
Albumin (g/dl)	**1.67** [[4], [5], [6]]	**0.05** (2.4–4.6)	**0.15** [[4], [5], [6]]	**0.51** [[4], [5], [6]]
Glucose (mg/dl)	**132.5** (70–100)	**158.3** (70–140)	**152.7** (70–100)	**122.3** (70–100)
LDH (U/L)	**308** (0–248)	**64** (225–450)	37 (0–248)	36 (0–248)
ADA (U/L)		5.7 (<26)	4 (<26)	
TLC		300	200	
Neutrophils		10% (30)	17	
Lymphocytes		**90% (270)**	**183**	

The serum ascites albumin gradient (SAAG) was >1.1 g/dl, not suggestive of TB pathology. Similarly, pleural and pericardial fluid did not fulfil the Light's criteria for exudative effusion [5]. But the lymphocytic predominance in ascitic and pleural fluid still suggested TB pathology in accordance with FNAC findings. Despite the dilemma of exudative or transudative polyserositis, diagnosis of disseminated TB was done and anti-TB therapy (ATT) was started with 2 months HRZE (Isoniazid, Rifampicin, Pyrazinamide and Ethambutol) plus 7–10 months HRE based on the national guideline [6]. Two pint of packed red blood cell (PRBC) was also transfused during the course of stay in hospital as her hemoglobin dropped down to 6.5 g/dl.

She accepted the treatment and was tolerating medications well. She was then discharged on ATT with counselling to have good nutrition and follow up on regular basis.

## 3 Discussion

Tuberculosis is one of the common infectious diseases globally. It stands among top ten causes of death [7]. Among different forms of TB, disseminated TB is one of the life-threatening conditions. Although exact epidemiology of disseminated TB is unclear, it is estimated to account less than 2% of all TB cases and up to 20% of extra-pulmonary cases [3]. The definition of disseminated TB involves hematogenous dissemination *Mycobacterium tuberculosis* in two or more non-contiguous sites. It can occur through primary focus, reactivation of latent TB (post-primary TB) or rarely through iatrogenic origin [1,3]. The occurrence of disseminated has been significantly shown with immunocompromised people by various studies [1,3,8,9]. The exact mechanism for dissemination is still unclear. However, dissemination of TB bacilli into the pulmonary vein through the erosion of epithelial layer of alveolar cell is one of the proposed mechanism for disseminated TB [3,10].

The disseminated TB presents with non-specific signs and symptoms based on the organs involved. It can range from anorexia, fever with chills and rigor, cough, weight loss, fatigue, lymphadenopathy, ascites, pleural and pericardial effusion and anemia [1,3]. The less common symptom our patient had was watery diarrhea which was common in children [3]. The involvement of lung, abdomen and heart as polyserositis in disseminated TB is rare as presented in our patient [4]. Polyserositis has been defined as inflammatory membranous thickening lining great serous sacs [11]. But as polyserositis can be the presentation in disseminated TB it can guide to the diagnosis of disseminated TB through fluid analysis [12,13]. The polyserositis due to TB is usually exudative. The analysis of ascitic fluid revealed SAAG >1.1 g/dl which was not in line of TB pathology. The liver pathology, especially portal hypertension secondary to liver cirrhosis was ruled out as the clinical and laboratory parameters for liver pathology were normal in our patient. Similarly, the pleural and pericardial fluid analysis based on Light's criteria was against the exudative effusion. But the cells analysis in pleural and ascitic fluid had lymphocytic predominance which was in the favor TB pathology.

The likely explanation for this kind of result is hypoalbuminemia, which was solely seen in all of the fluid analysis. The link between tuberculosis and nutrition has been studied for many years. The effect of tuberculosis on nutritional state has negative impact with abnormal protein metabolism, so-called anabolic block, due to abnormal cytokine activation [14,15]. Low albumin level is the consequence of altered nutritional status due to tuberculosis [16,17]. Hypoalbuminemia has also been found as the predictive factor for prognosis in TB patients [16]. Hence, as in our case the transudative nature of the fluid was likely due to hypoalbuminemia resulting from malnutrition due to TB. The lymphocytic predominance and histopathological evidence furthermore confirmed the diagnosis of disseminated TB.

Polyserositis in disseminated TB is itself an uncommon presentation. In addition to it, the confusing nature of polyserositis makes the diagnosis of disseminated tuberculosis difficult. There have been very few mentions of polyserositis in disseminated TB. And the clear picture of the polyserositis has not been well-explained in the existing literature. Though TB effusions are usually exudative but can be presented as transudative as in our case. This does not mean TB effusions are transudative. Rather it should have clear understanding and explanation.

We could not do follow up with our patient after she was discharged from the hospital. Similarly, 2D echocardiography image could not be presented to show pericardial effusion. These remain as our limitation during the study.

## 4 Conclusions

Disseminated TB is itself a diagnostic challenge for clinicians. The polyserositis with transudative nature, as present in our patient, diverts away from our current understanding of TB. However, it also gives us the opportunity to think about the possible mechanism. As hypoalbuminemia due to TB was the reason behind transudative nature of the polyserositis in our case of disseminated TB. It adds to the current understanding of polyserositis in disseminated TB that variations are possible and should be sought for.

## 5 Future perspectives

Although tuberculosis is one of the most prevalent diseases in the world there are still loop holes that need to be searched for better understanding of the disease. Some of the future perspectives in which research should be thoroughly conducted are proteomic biomarkers for rapid diagnosis of TB and newer anti-TB drugs for the treatment of multi-drug and extensive-drug resistant TB.

## Ethical approval

This is a case report, therefore, it did not require ethical approval from ethics committee.

## Funding

The study did not receive any grant from funding agencies in the public, commercial or not-for-profit sectors.

## Author contributions

We the undersigned declare that this manuscript is original, has not been published before and is not currently being considered for publication elsewhere.

We confirm that the manuscript has been read and approved by all named authors and that there are no other persons who satisfied the criteria for authorship but are not listed. We further confirm that the order of authors listed in the manuscript has been approved by all of us.

We understand that the Corresponding Author is the sole contact for the Editorial process. He/she is responsible for communicating with the other authors about progress, submissions of revisions and final approval of proofs.

**Correspondence:** Bishal Dhakal, Nepalese Army Institute of Health and Sciences, 44600 Kathmandu, Nepal. Email: swarnimdhakal@gmail.com, Phone: +977 9846491651.

Authors as follows:

1. Bishal Dhakal.

2. Prabhat K.C.

3. Sachin Sapkota.

4. Binaya Subedi.

5. Apshara Acharya.

6. Suvekchya Pandey.

7. Dilip Thapa.

Author 1: Contributed in data collection, literature review, writing the manuscript.

Author 2: The resident physician, who helped in the diagnosis and supervised in case presentation.

Author 3: Contributed in supervision and editing the manuscript.

Author 4: Contributed in manuscript review and data collection.

Author 5: Contributed in data collection and literature review.

Author 6: Contributed to supervision, conceptualization and data collection.

Author 7: Contributed in data collection and literature review.

## Registration of research studies

Not applicable.

## Consent

Written informed consent was obtained from the patient for publication of this case report and accompanying images. A copy of the written consent is available for review by the editor-in-chief of this journal on request.

## Guarantor

Bishal Dhakal, Nepalese Army Institute of Health and Sciences, 44600 Kathmandu, Nepal. Email: swarnimdhakal@gmail.com, Phone: +977 9846491651.

## Provenance and peer review

Not commissioned, externally peer reviewed.

## Declaration of competing interest

The authors report no conflicts of interest.
