# Supplementary material for: Transudative or masked exudative polyserositis in disseminated tuberculosis? A case report
Source: Ann Med Surg (Lond). 2022 May 31;78:103891. doi: 10.1016/j.amsu.2022.103891 (PMC9207084; doi:10.1016/j.amsu.2022.103891)
Supplement: Multimedia component 1 [file mmc1.docx]

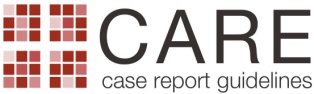
CARE Checklist of information to include when writing a case report
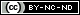


**Topic Item Checklist item description Reported**

**Title 1** The diagnosis or intervention of primary focus followed by the words “case report” Yes

**Key Words 2** 2 to 5 key words that identify diagnoses or interventions in this case report, including "case report" Yes

Abstract

**(no references)**

**3a** Introduction: What is unique about this case and what does it add to the scientific literature? Yes

**3b** Main symptoms and/or important clinical findings Yes

**3c** The main diagnoses, therapeutic interventions, and outcomes Yes

**3d** Conclusion—What is the main “take-away” lesson(s) from this case? Yes

**Introduction 4** One or two paragraphs summarizing why this case is unique (**may include references**) Yes

**Patient Information 5a** De-identified patient specific information Yes

**5b** Primary concerns and symptoms of the patient Yes

**5c** Medical, family, and psycho-social history including relevant genetic information -

**5d** Relevant past interventions with outcomes -

Clinical Findings

**Timeline**

**Diagnostic Assessment**

**Therapeutic Intervention**

**Follow-up and Outcomes**

1. Describe significant physical examination (PE) and important clinical findings Yes
2. Historical and current information from this episode of care organized as a timeline Yes

**8a** Diagnostic testing (such as PE, laboratory testing, imaging, surveys). Yes

**8b** Diagnostic challenges (such as access to testing, financial, or cultural) -

**8c** Diagnosis (including other diagnoses considered) Yes

**8d** Prognosis (such as staging in oncology) where applicable -

**9a** Types of therapeutic intervention (such as pharmacologic, surgical, preventive, self-care) Yes

**9b** Administration of therapeutic intervention (such as dosage, strength, duration) Yes

**9c** Changes in therapeutic intervention (with rationale) P9-10

**10a** Clinician and patient-assessed outcomes (if available) -

**10b** Important follow-up diagnostic and other test results -

**10c** Intervention adherence and tolerability (How was this assessed?) -

**10d** Adverse and unanticipated events -

**Discussion 11a** A scientific discussion of the strengths AND limitations associated with this case report Yes

**11b** Discussion of the relevant medical literature **with references** Yes

**11c** The scientific rationale for any conclusions (including assessment of possible causes) Yes

**11d** The primary “take-away” lessons of this case report (without references) in a one paragraph conclusion Yes

**Patient Perspective 12** The patient should share their perspective in one to two paragraphs on the treatment(s) they received -

**Informed Consent 13** Did the patient give informed consent? Please provide if requested . . . . . . . . . . . . . . . . . . . . . . . . . . . . . . . . . . . . . . **Yes √ No**
